# Obsessive-Compulsive Personality Disorder and Death by Suicide

**DOI:** 10.1192/j.eurpsy.2023.518

**Published:** 2023-07-19

**Authors:** J. Overholser, S. Gomez, C. McGovern, C. Silva, C. Stockmeier

**Affiliations:** ^1^Psychological Sciences, Case Western Reserve University, Cleveland; ^2^Psychiatry, University of Mississippi, Jackson, United States

## Abstract

**Introduction:**

The risk of suicide may be elevated in the presence of personality pathology. Adults with Obsessive Compulsive Personality Disorder (OCPD) may be vulnerable to depression and suicidal thoughts.

**Objectives:**

To identify factors associated with suicide in cases of OCPD.

**Methods:**

Psychological autopsy procedures were used to gather detailed information about adults who died by suicide and natural causes. A total of 75 deceased adults were evaluated using psychological autopsy procedures. Family members were interviewed about a recently deceased adult, using structured diagnostic interviews (SCID and SIDP-IV). Diagnostic summaries, coroner’s reports and police records were reviewed by a psychiatrist, a psychologist, a social worker, and a neuroscientist until agreement was reached about final diagnosis. The final sample included 40 adults who met criteria for OCPD (18 had died by suicide; 20 had died by natural causes). An additional 40 cases were examined in which evidence of PD was absent (19 had died by suicide; 18 had died by natural causes).

**Results:**

The diagnosis of a Major Depressive Disorder was significantly more common in suicide completers with OCPD compared to suicide completers without OCPD (***X***^2^ = 6.74, p < .01) or cases of natural death with OCPD (***X***^2^ = 12.70, p < .001). Suicide completers with OCPD displayed many symptoms of depression, more often than suicide completers without OCPD or cases of natural death with OCPD (see Table 1). As compared to the cases of natural death, both groups of suicide completers were more likely to have previously attempted suicide prior to their final act (***X***^2^ = 8.52, p < .05).

**Table 1.** Comparison of four groups using psychological autopsy procedures to identify the presence of diagnostic criteria for a Major Depressive Episode at the time of death.

	OCPD Suicide	OCPD Natural Death	No PD Suicide	No PD Natural Death	***X***^2^
Sad mood	82.4%	36.8%	78.9%	50.0%	11.38 **
Sleep disturbance	82.4%	38.9%	73.7%	46.7%	9.53 *
Feelings of worthlessness	60.0%	38.9%	84.2%	17.6%	17.49 ***
Reduced concentration	58.8%	27.8%	57.9%	14.3%	9.89 *
Recurrent suicidal ideation	88.2%	26.3%	78.9%	0.0%	35.57 ***
Loss of pleasure	82.4%	38.9%	73.7%	40.0%	10.80 **
Psychomotor changes	50.0%	33.3%	61.1%	26.7%	5.04
Reduced energy	64.7%	44.4%	63.2%	33.3%	4.12
Change of appetite	70.6%	26.3%	42.1%	31.3%	8.37 *

Note:

* = p < .05;

** = p < .01;

*** = p < .001

**Conclusions:**

Adults with OCPD appear vulnerable to a Major Depressive episode, and the combination of MDD with OCPD creates a significant risk for death by suicide. It is important to appreciate the influence of personality disorder or depression and suicide risk.

**Disclosure of Interest:**

None Declared

